# Supplementary material for: Transient Exposure of Endothelial Cells to Doxorubicin Leads to Long-Lasting Vascular Endothelial Growth Factor Receptor 2 Downregulation
Source: Cells. 2022 Jan 8;11(2):210. doi: 10.3390/cells11020210 (PMC8773916; doi:10.3390/cells11020210)
Supplement: Supplementary file 1 [file cells-11-00210-s001.zip › cells-1534289-supplementary.pdf]

SUPPLEMENTAL MATERIAL

Supplementary figure S1

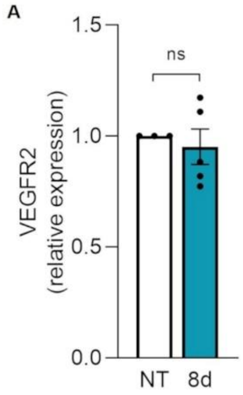

Figure S1. VEGFR2 expression during Dox-induced senescence. HUVECs were incubated with 250nM Doxorubicin for 24h. After extensive washing the medium was replaced with free-drug complete medium and cells were maintained in culture for additional days. Samples were collected and analysed after 8 days from the removal of the drug (8d), blue histogram. NT= not treated cells, white histogram. mRNA level of VEGFR2 determined by qRT-PCR. Each sample was normalized to β-tubulin and expressed as fold increase compared to NT. Data are shown as mean ± SEM of n=3-5 independent experiments per condition. ns $p > 0.05$ by unpaired Student's *t* test.

Supplementary figure S2

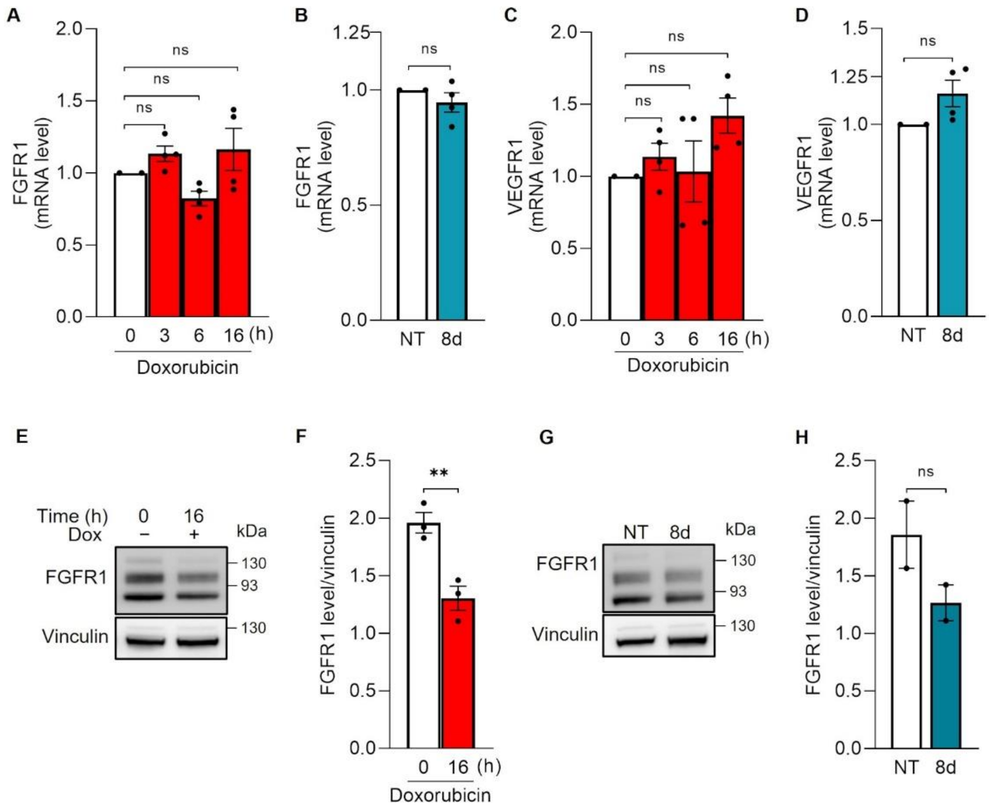

Figure S2. Effect of Dox on FGFR1 and VEGFR1 levels. HUVECs were incubated with 250nM Doxorubicin and samples were collected at the indicated time during the treatment (red histogram). White histogram represents the not treated cells, blue histogram refers to dox-induced senescent cells at 8 days after drug removal. (**A-D**) mRNA level of FGFR1 and VEGFR1 determined by qRT-PCR. Each sample was normalized to β-tubulin and data expressed as fold increased compared to untreated sample. Data are shown as mean ± SEM of n=2-4 independent experiments per condition. ns p >0.5 by unpaired Student's *t* test. (**E-H**) Cell lysates analysed by immunoblotting with FGFR1 antibody. (**E**,**G**) Representatives immunoblots (**F,H**) Quantification of FGFR1 by densitometric analysis. Data are shown as mean ± SEM of FGFR1 protein level normalized to vinculin. n=2-3 independent experiments per condition. ns p>0.05, **p <0.01 by unpaired Student's *t* test.

Supplementary figure S3

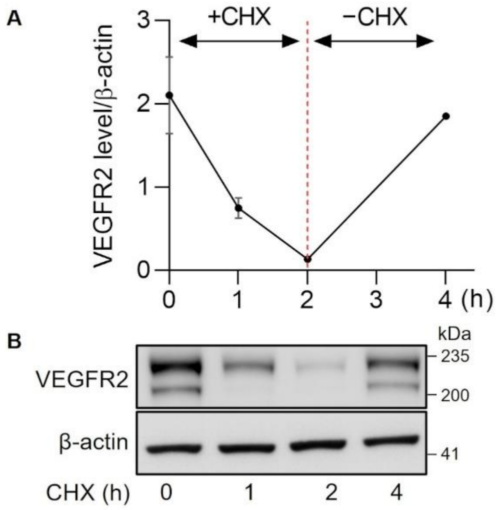

Figure S3. VEGFR2 turnover in normal growth conditions. HUVECs were incubated with 10µg/ml Cycloheximide (CHX) up to 2h. After extensive washing, the medium was replaced with free-drug complete medium, and cells were maintained in culture for additional 2h. Cell were lysed at the indicated time points and analysed by immunoblotting with VEGFR2 antibodies **(A-B)**. Representative immunoblot (B) and relative quantification (A) of VEGFR2 protein level by densitometric analysis. Data are shown as mean ± SEM of protein level normalized to β-actin. n=2 independent experiments per condition.

Supplement figure S4

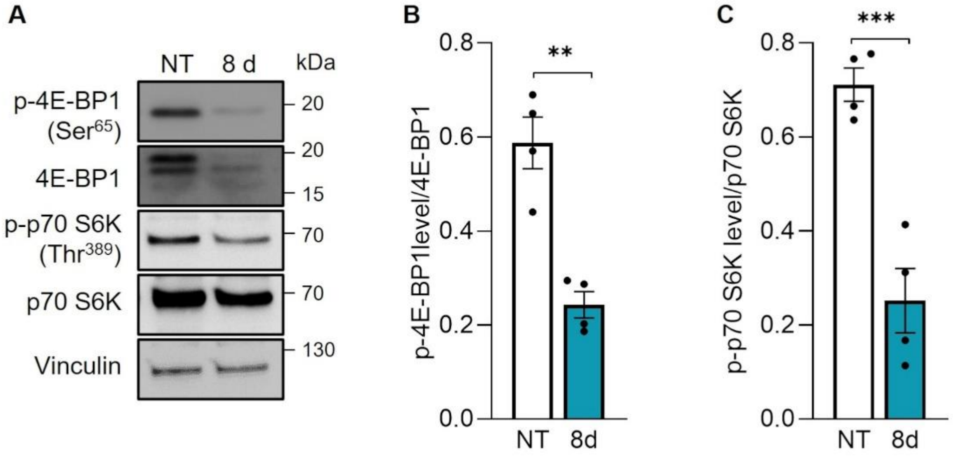

Figure S4. mTOR activity during Dox-induced senescence. **(A-C)** HUVECs were incubated with 250nM Doxorubicin for 24h. After extensive washing the medium was replaced with free-drug complete medium and cells were maintained in culture for additional days. Samples were collected and analysed after 8 days from the removal of the drug (8d), blue histogram, by immunoblotting with the indicated antibodies for mTORC1 signalling. NT= not treated cells, white histogram. **(A)** Representative immunoblot. **(B-C)** Quantification of p-4E-BP1 (Ser^{65} and total) and p-p70 S6K (Thr^{389} and total), respectively, by densitometric analysis. Data are shown as mean ± SEM of phosphorylated protein level normalized to total protein level. n=4 independent experiments per condition. **p <0.01, ***p <0.001 by unpaired Student's *t* test.
